# Dissection and Fine-Mapping of Two QTL Controlling Grain Size Linked in a 515.6-kb Region on Chromosome 10 of Rice

**DOI:** 10.3390/plants13152054

**Published:** 2024-07-25

**Authors:** Yi Shen, Derun Huang, Zhenhua Zhang, Yeyang Fan, Zhonghua Sheng, Jieyun Zhuang, Bo Shen, Yujun Zhu

**Affiliations:** 1 Jiangxi Early-Season Rice Research Centre, China National Rice Research Institute, Hangzhou 310006, China; shengzhonghua@caas.cn; 2 College of Life and Environmental Sciences, Hangzhou Normal University, Hangzhou 310012, China; yishen1912@163.com; 3 State Key Laboratory of Rice Biology and Breeding, China National Rice Research Institute, Hangzhou 310006, China; huangderun@caas.cn (D.H.); zhangzhenhua@caas.cn (Z.Z.); fanyeyang@caas.cn (Y.F.); zhuangjieyun@caas.cn (J.Z.)

**Keywords:** rice, grain weight, grain size, minor effect, closely linked, quantitative trait locus

## Abstract

Grain size is a primary determinant of grain weight, which is one of the three essential components of rice grain yield. Mining the genes that control grain size plays an important role in analyzing the regulation mechanism of grain size and improving grain appearance quality. In this study, two closely linked quantitative trait loci (QTL) controlling grain size, were dissected and fine-mapped in a 515.6-kb region on the long arm of chromosome 10 by using six near isogenic line populations. One of them, *qGS10.2*, which controlled 1000 grain weight (TGW) and grain width (GW), was delimited into a 68.1-kb region containing 14 annotated genes. The Teqing allele increased TGW and GW by 0.17 g and 0.011 mm with the *R*^2^ of 12.7% and 11.8%, respectively. The other one, *qGL10.2*, which controlled grain length (GL), was delimited into a 137.3-kb region containing 22 annotated genes. The IRBB52 allele increased GL by 0.018 mm with the *R*^2^ of 6.8%. Identification of these two QTL provides candidate regions for cloning of grain size genes.

## 1. Introduction

Grain size is a primary determinant of grain weight, which is one of the three essential components of rice grain yield. Additionally, grain size is also a trait for grain appearance quality that rice breeders pay attention to, because the rice consumers in many countries prefer slender rice. Mining the genes that control grain size plays an important role in analyzing the regulation mechanism of grain size and improving grain appearance quality.

To date, causal genes for 28 QTL controlling grain size have been cloned [1,2,3,4], including genes for 1000-grain weight (TGW) such as *qTGW1.2b* [4], *TGW2* [5], *qTGW3*/*GL3.3* [6,7] and *TGW6* [8]; genes for grain length (GL) such as *GS3* [9], *GSA1* [10], *GL3.1*/*qGL3* [11,12], *qGL5* [13] and *GL6* [14]; and genes for grain width (GW) such as *GW2* [15], *GW5*/*GSE5* [16,17], *GW6* [18] and *GW8* [19]. These cloned QTL have been found to be involved in several signal pathways regulating cell elongation and proliferation, such as plant hormone signaling, G-protein signaling, ubiquitin-proteasome pathway, and a range of transcriptional regulators. Eleven of them, *GS2*/*GL2* [20,21], *GL3.1*/*qGL3* [11,12], *GS3.1* [22], *GSW3* [3], *qTGW3*/*GL3.3* [6,7], *GS5* [23], *GW5*/*GSE5* [16,17], *qGL5* [13], *GW6* [18], *TGW6* [8] and *GW10* [24], encoded components of Brassinosteroid, Gibberellin and Auxin signaling pathways. Ten of them, *OsLG3* [25], *SG3* [26], *OsLG3b*/*qLGY3* [27,28], *GW6a* [29], *GL6* [14], *GL7*/*GW7* [30,31], *GLW7* [32], *GW8* [19], *GS9*/*GL9* [33,34] and *qTGW10-20.8*/*qGL10*/*GL10* [35,36,37], encoded transcriptional regulatory factors. Two of them, *GW2* [15] and *GS3* [9], were involved in the ubiquitin-proteasome and G-protein signaling pathways, respectively. One of them, *GSA1* [10], regulating grain size and abiotic stress tolerance, was involved in metabolic flux redirection. The remaining four, *qTGW1.2b* [4], *OsPUB3* [38,39], *TGW2* [5] and *GSE9* [3], were not clear about the regulatory pathways involved at present. These studies have gradually elucidated the regulatory pathways of rice grain size, providing valuable references for mining new genes and unraveling regulatory mechanisms.

Although there were many QTL cloned for grain size, their proportion was still very low compared to the number of QTL that have been primary mapped. According to statistics, a total of 568 QTL for grain size were collected in Gramene database (https://archive.gramene.org/qtl/, accessed on 15 April 2024), and only 4.9% of them were cloned. The reason was that most of these cloned QTL show major effects and were easy to be isolated and cloned. However, the genetic effects of most QTL for grain size were small, and the phenotype was easily disturbed by environment and background genes, which made it difficult for fine-mapping and gene functional complementation verification. Nonetheless, based on quantitative genetics theory and modern molecular mapping results, minor-effect QTL also played important roles in regulating important agronomic traits in rice, whether in mechanism analysis or breeding applications [40], these QTL cannot be ignored.

In our previous study, a minor-effect QTL for controlling TGW and GW, *qGS10.2*, was located in the region RM3123–RM6673 on the long arm of chromosome 10 by using five near isogenic line (NIL) populations derived from the cross between *indica* rice varieties Teqing (TQ) and IRBB52 [41]. In this study, the genetic effect of *qGS10.2* on grain size was further validated by using six NIL populations. Finally, two closely linked QTL controlling grain size, were dissected and fine-mapped in a 515.6-kb region. *qGS10.2*, which controlled TGW and GW, was delimited into a 68.1-kb region. *qGL10.2*, which controlled GL, was delimited into a 137.3-kb region.

## 2. Results

### 2.1. Validation of qGS10.2

**Figure 1:**
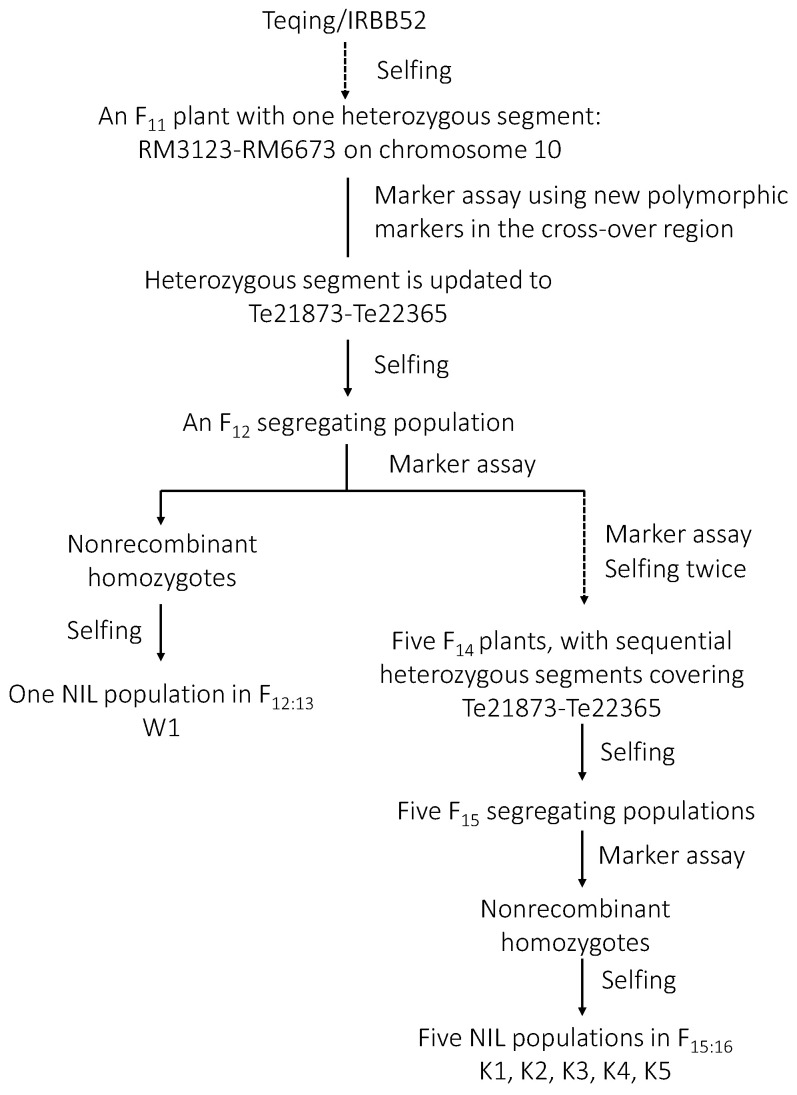
Development of the rice populations used in this study. NIL, near isogenic line.

One F_12:13_ NIL population carrying the heterozygous region Te21873–Te22365, W1, was firstly used to validate the genetic effect of *qGS10.2*. As illustrated in Figure 1, it consisted of 28 NIL-TQ homozygous lines and 28 NIL-IRBB52 homozygous lines in the segregating region, derived from an F_11_ plant of the rice cross TQ/IRBB52. At maturity, three traits, TGW, GL and GW, were measured.

**Figure 2:**
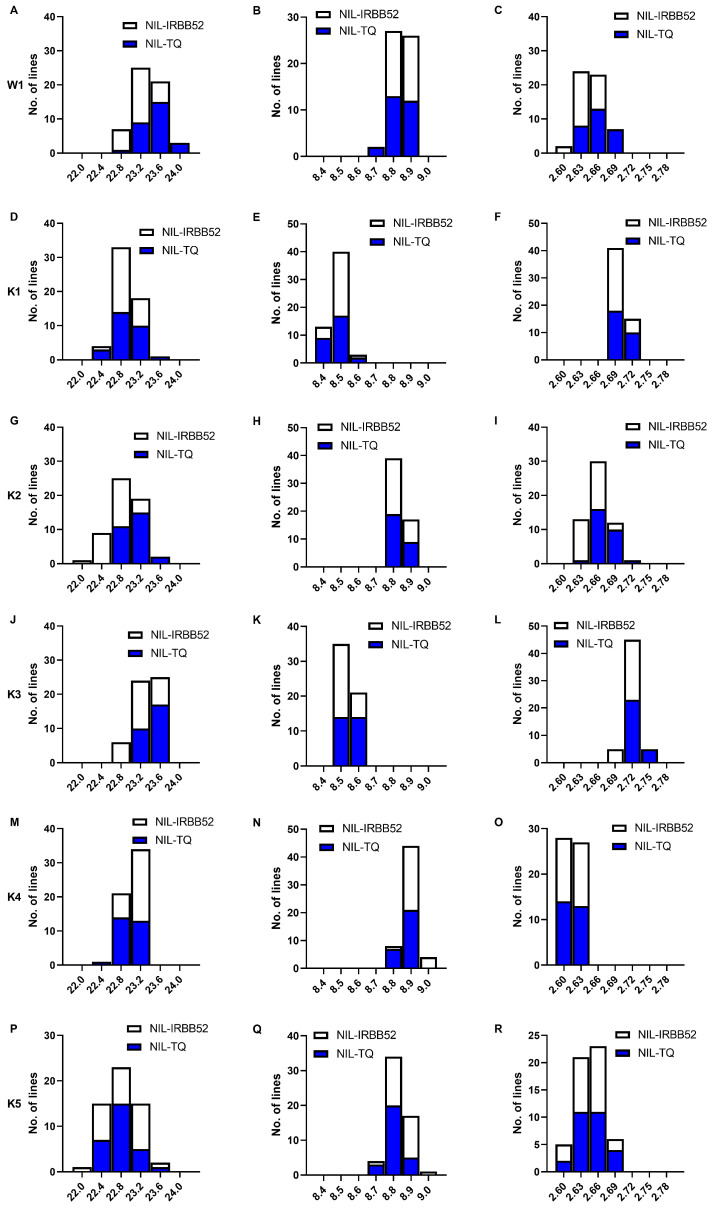
Distributions of 1000 grain weight, grain length and grain width in six near isogenic line populations. (**A**–**C**): W1, (**D**–**F**): K1, (**G**–**I**): K2, (**J**–**L**): K3, (**M**–**O**): K4, (**P**–**R**): K5. TGW, 1000 grain weight; GL, grain length; GW, grain width; NIL-TQ and NIL-IRBB52 are near isogenic lines having Teqing and IRBB52 homozygous genotypes in segregating region, respectively.

Two genotypic groups were used as two series to plot the frequency distributions of the three traits. For TGW and GW, the NIL-TQ lines concentrated in the high-value area, and the NIL-IRBB52 lines concentrated in the low-value area (Figure 2A,C). These results suggested that QTL for TGW and GW was segregated in W1 with the enhancing allele derived from TQ.

**Figure 3:**
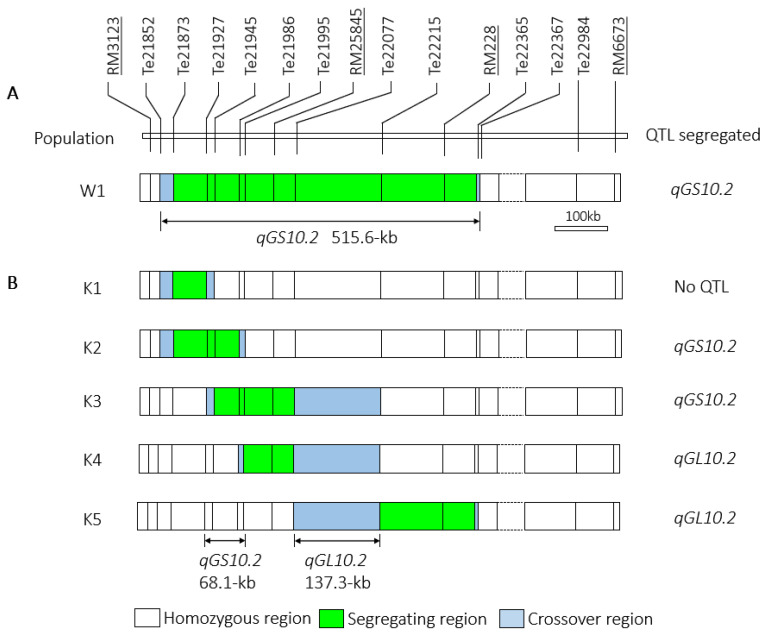
Segregating regions of the six near isogenic line populations. (**A**) W1 was used to validate the genetic effect of *qGS10.2*. The underlined molecular markers were used in previous study. (**B**) Five populations were used for fine-mapping of *qGS10.2*.

Two-way analysis of variance (ANOVA) was performed to test the phenotypic differences in W1 population. The analysis was performed using the statistical analysis software SAS [42]. A mixed model GENOTYPE + LINE (GENOTYPE) + REP + GENOTYPE*REP was applied, in which LINE (GENOTYPE) was defined as a random effect and used as the error term to test GENOTYPE differences. When significant differences were detected (*p* < 0.05), the additive effect was estimated by (IRBB52–TQ)/2. Positive values indicate that the increasing allele from IRBB52, and negative values indicate that the increasing allele from TQ. As shown in Table 1, highly significant effects were detected for TGW and GW. The TQ allele increased TGW and GW by 0.14 g and 0.011 mm with the *R*^2^ of 6.6% and 8.1%, respectively. The results indicated that *qGS10.2* was segregated in the region flanked by markers Te21852 and Te22367 (Figure 3A), corresponding to a 531.6-kb region in the Nipponbare genome.

### 2.2. Dissection and Fine-Mapping of qGS10.2

Five F_15:16_ NIL populations, heterozygous in Te21873–Te21927, Te21873–Te21986, Te21945–Te22077, Te21995–Te22077, and Te22215–Te22365, respectively, were used to fine-mapped *qGS10.2* (Figure 3B). They were derived from five F_14_ plants with sequential heterozygous segments covering the region Te21873–Te22365 (Figure 1). Each of the NIL population consisted of 28 NIL-TQ homozygous lines and 28 NIL-IRBB52 homozygous lines in the segregating regions.

Frequency distributions of the three traits in each population were exhibited in Figure 2D–R. In K2 and K3, the NIL-TQ and NIL-IRBB52 lines concentrated in the high- and low-value areas of TGW and GW, respectively (Figure 2G–L). In K4 and K5, the NIL-IRBB52 and NIL-TQ lines concentrated in the high- and low-value areas of GL, respectively (Figure 2M–R). These results suggested that there may be two QTL segregating in the region of qGS10.2, one controlled TGW and GW, and the other controlled GL.

Results of the two-way ANOVA on the three traits in these five NIL populations were shown in Table 1. No significant effect was detected in K1. Highly significant effects were detected for TGW and GW in K2 and K3. Additive effects estimated in these two NIL populations were similar. The TQ allele increased TGW and GW by 0.17 and 0.13 g, and 0.011 and 0.007 mm with the *R^2^* of 12.7% and 10.0%, 11.8% and 10.7%, respectively. In addition, highly significant effects were also detected for GL in K4 and K5. The additive effects estimated in these two populations were also similar. The IRBB52 allele increased GL by 0.018 and 0.017 mm with the *R^2^* of 6.8% and 6.7%, respectively.

As shown in Figure 3B, since the segregating regions of K2 and K5 were totally separated from each other, this suggested that each of these two segregating regions contained one QTL. The additive effects of TGW and GW detected in K2 and K3 were similar to those of *qGS10.2*, indicating that *qGS10.2* was located in the common segregating region of K2 and K3. This region was flanked by markers Te21927 and Te21995, corresponding to a 68.1-kb region in the Nipponbare genome. In addition, the other QTL detected in K4 and K5, suggesting that this QTL was located in the common segregating regions of K4 and K5. This region was flanked by markers Te22077 and Te22215, corresponding to 137.7-kb region in the Nipponbare genome. Since this QTL only controlled GL, it is named *qGL10.2*.

In summary, two QTL closely linked in a 515.6-kb region were separated. The *qGS10.2* controlling TGW and GW was delimited into a 68.1-kb region. The other QTL, *qGL10.2* controlling GL, was located within a 137.3-kb region.

### 2.3. Candidate Genes for qGS10.2 and qGL10.2

According to the Rice Genome Annotation Project (http://rice.uga.edu/, accessed on 30 April 2024), there are fourteen annotated genes in the 68.1-kb region of *qGS10.2*. As shown in Table 2, three annotated genes encode retrotransposon protein, two encode expressed protein, one encodes a hypothetical protein, and the remaining eight encode protein with known functional domains. Three of the eight, *Os10g40880*, *Os10g40900* and *Os10g40934*, all encode flavonol synthases. The other five, *Os10g40810*, *Os10g40830*, *Os10g40859*, *Os10g40920* and *Os10g40950*, encode GATA zinc finger domain containing protein, metalloendoproteinase 1 precursor, matrixin family protein, pentatricopeptide and polyol transporter 5, respectively.

For the *qGL10.2*, there are 22 annotated genes in the 137.3-kb region (Table 2). One of them encodes a hypothetical protein, six of them encode expressed protein, and the remaining 15 encode protein with known functional domains. Four of the fifteen, *Os10g41130*, *Os10g41200*, *Os10g41260* and *Os10g41330*, encode AP2 domain containing protein and MYB family transcription factor, which are similar to the proteins encoded by the cloned grain size genes *OsLG3* [25] and *SG3* [26]. In addition, the coding products of the other 11 annotation genes are different from those of the cloned grain size genes. More studies will continue for map-based cloning of *qGS10.2* and *qGL10.2*.

## 3. Discussion

In this study, we identified two closely linked QTL controlling grain size from a 515.6-kb region on the long arm of chromosome 10. One QTL controlling TGW and GW, *qGS10.2*, was delimited into a 68.1-kb region, which contained 14 annotated genes. One of them, *Os10g40810* encoding a GATA zinc finger domain containing protein, functions in controlling rice plant architecture and panicle/grain development. The knock-down lines in a *japonica* variety Wuyunjing 7 background have ideal architecture, better grain shape, and enhanced grain yield [43]. These results implied that *Os10g40810* was the most likely candidate gene for *qGS10.2*. The other one controlling GL, *qGL10.2*, was delimited into a 137.3-kb region, which contained 22 annotated genes. In addition, we also noticed that the GL of NIL-TQ lines was longer than that of NIL-IRBB52 lines in K2 and K3 populations separated by *qGS10.2* (Table 1). This result showed that TQ allele may have an effect of increasing GL in *qGS10.2* segregation region, but it was not statistically significant. This may be the reason why the genetic effect of *qGL10.2* was not detected in W1 population, that is, TQ allele increased the GL in *qGS10.2* region, but decreased the GL in *qGL10.2* region. In the meantime, it was found that K1 and K3 differed from the remaining three populations in terms of the GL and GW (Table 1), which was not unexpected since K2, K4 and K5 were derived from the same F_12_ recombinant plant, whereas K1 and K3 were derived from another F_12_ recombinant plant. This phenomenon also happened in our previous study [44]. Identification of these two QTL provides candidate regions for cloning of grain size genes.

In our previous study, three QTL controlling grain size, *qGL10*, *qGS10.1* and *qGS10.2*, were identified in a 4.2-Mb region on the long arm of chromosome 10 [41]. Up to now, *GW10* controlling grain size and number [24] and *qTGW10-20.8*/*qGL10*/*GL10* [35,36,37] controlling TGW and GL have been cloned in the region of *qGL10* and *qGS10.1*, respectively. In this study, we not only fine-mapped *qGS10.2*, but also identified a new QTL, *qGL10.2*, controlling grain length. For the region of *qGS10.2*, one annotated gene, *Os10g40810*, was determined to be related to rice grain development by reverse genetics [43]. It suggested that there were at least three genes controlling grain size in the 4.2-Mb region. Similarly, the phenomenon of tightly linked genes simultaneously controlling grain size also occurs on the other chromosomes. *OsLG3* and *OsLG3b* for grain length were cloned in a 1.7-Mb region on chromosome 3 [25,27,28], *GS5* and *GSE5* for grain width in a 1.9-Mb region on chromosome 5 [16,17,23], and *TGW6* and *GW6a* for grain weight in a 0.6-Mb region on chromosome 6 [8,29]. These results implied that the genes controlling quantitative traits are often distributed in clusters.

In recent years, our group has cloned several minor-effect QTL controlling grain size, such as *qTGW1.2b*, *qGS1-35.5* and *qTGW10-20.8* [4,35,38]. In the process of map-based cloning of these QTL, a genetic resource, residual heterozygote (RH), was used for the construction of NIL populations. RH means that a single plant was heterozygous in the region covering the target QTL, while in other regions, it was homozygous for the parental alleles. The advantage was that the NIL populations derived from a RH can effectively eliminate the interference of background genes. Therefore, although the genetic effects of the target QTL were small, the effects remain stable in different NIL populations. For example, the additive effects of *qGS10.2* and *qGL10.2* here were similar in K2 and K3, K4 and K5 populations respectively (Table 1). Based on this advantage, more and more studies began to apply RH to map-based cloning of QTL for yield-related traits in rice [45,46].

Generally, the QTL genetic effects are determined by the phenotypic differences between different alleles in a genetical population. For example, if the two parents carry functional and non-functional alleles respectively at the target gene locus in a bi-parental mapping population, the genetic effect is generally large. Conversely, if the two parents both carry functional alleles, the effect may be small. *qTGW10-20.8*/*OsMADS56* is a minor-effect QTL for controlling grain weight and grain length, which was previously cloned from the cross between *indica* rice varieties TQ and IRBB52 by our group [35]. Since both TQ and IRBB52 carrying functional alleles, genetic effect analysis reveals *qTGW10-20.8* as a minor effect QTL, with TQ allele increasing TGW and GL by only 0.22 g and 0.026 mm, respectively. In addition, this gene was also isolated from two other *indica*/*japonica* crosses, Huajingxian 74 (HJX74) / Lemont and Zhai-Ye-Xi (ZYX) / 02428 [36,37]. Compared with the genome sequence of HJX74, Lemont has a 1019-bp deletion in the 5′UTR and exon 1 region, resulting in the loss of transcriptional activity of *OsMADS56* [36]. In another cross, five haplotypes were detected from 15 SNPs in the *OsMADS56* promoter region. The Hap3 was associated with higher values of TGW and GL and was distributed mainly in the *japonica* varieties [37]. Due to the genetic effects of *OsMADS56* representing the difference between the *indica*- and the *japonica*-allele in these two mapping populations, it is manifested as a major effect gene. These results suggested that strong functional allele can be discovered from germplasm resources through minor-effect gene cloning and haplotype analysis.

## 4. Materials and Methods

### 4.1. Rice Materials

Six NIL populations segregating in an isogenic background were used in this study. As shown in Figure 1, one F_11_ plant of TQ/IRBB52, heterozygous in the region RM3123–RM6673, was firstly selected. Then, 11 new polymorphic markers were developed in this region and used to test genotypes of the F_11_ plant. The heterozygous region was updated to Te21873–Te22365. In the resultant F_12_ population consisting of 192 plants, homozygous non-recombinants were identified and selfed to develop homozygous lines. One NIL population in F_12:13_ named W1 was constructed and was used for validation of *qGS10.2*.

In addition, five F_14_ plants, heterozygous in Te21873–Te21927, Te21873–Te21986, Te21945–Te22077, Te21995–Te22077, and Te22215–Te22365, respectively, were identified and selfed to produce five F_15_ populations. In each population, homozygous non-recombinants were identified and selfed to develop homozygous lines. Five F_15:16_ NIL populations named K1, K2, K3, K4 and K5 were constructed and were used for fine-mapping of *qGS10.2*.

### 4.2. Field Experiments and Phenotyping

All the NIL populations were planted in the paddy field of the China National Rice Research Institute in Hangzhou, Zhejiang province, China. For all trails, a randomized complete block design with two replications was performed. In each replication, each line was planted in a single row of 10 plants, with 26.7 cm between rows and 16.7 cm between plants. Field management followed local agricultural practice.

At maturity, four of the middle 8 plants in each row were harvested. Fully filled grains were selected and measured for TGW, GW and GL following the method reported by Zhang et al. [47].

### 4.3. DNA Marker Analysis

A total of 15 markers were used in this study, including 11 InDel and 4 simple sequence repeat (SSR) markers (Table S1). The InDels were developed based on the variance between TQ and IRBB52 as defined by whole-genome resequencing, while the SSRs were chosen from the Gramene database. DNA extraction and PCR amplification were followed Zheng et al. [48] and Chen et al. [49], respectively. The PCR products were visualized on 6.0% non-denaturing polyacrylamide gels using silver staining.

### 4.4. Data Analysis

For the six NIL populations, two-way ANOVA was used to analyze phenotypic differences between the two homozygous genotypic groups in each population. The analysis was performed using SAS procedure GLM (general linear model). When significant differences were detected (*p* < 0.05), the genetic effects of the QTL, including additive effect (*A*) and the proportions of phenotypic variance explained (*R*^2^) were estimated using the same model.

## 5. Conclusions

In this study, two closely linked QTL controlling grain size, were dissected and fine-mapped in a 515.6-kb region on the long arm of chromosome 10. One of them, *qGS10.2*, which controlled TGW and GW, was delimited into a 68.1-kb region. The other was *qGL10.2*, which controlled GL. It was delimited into a 137.3-kb region. Identification of these two QTL provides candidate regions for cloning of grain size genes.

## Figures and Tables

**Table 1:** Validation and fine-mapping of *qGS10.2* using six NIL populations.

Name	Segregating Region	Trait ^a^	Phenotypic Mean		*p*	*A* ^b^	*R*^2^ (%) ^c^
			NIL-TQ	NIL-IRBB52			
W1	Te21873-Te22365	TGW	23.47 ± 0.29	23.19 ± 0.19	<0.0001	−0.14	6.6
		GL	8.839 ± 0.055	8.847 ± 0.036	0.5400		
		GW	2.660 ± 0.020	2.637 ± 0.015	<0.0001	−0.011	8.1
K1	Te21873-Te21927	TGW	22.91 ± 0.25	22.89 ± 0.19	0.8001		
		GL	8.481 ± 0.040	8.491 ± 0.041	0.3785		
		GW	2.701 ± 0.013	2.700 ± 0.012	0.6483		
K2	Te21873-Te21986	TGW	23.06 ± 0.25	22.72 ± 0.31	<0.0001	−0.17	12.7
		GL	8.832 ± 0.045	8.813 ± 0.042	0.1160		
		GW	2.673 ± 0.016	2.651 ± 0.017	<0.0001	−0.011	11.8
K3	Te21945-Te22077	TGW	23.47 ± 0.20	23.22 ± 0.23	<0.0001	−0.13	10.0
		GL	8.540 ± 0.032	8.524 ± 0.041	0.1040		
		GW	2.727 ± 0.012	2.714 ± 0.013	<0.0001	−0.007	10.7
K4	Te21995-Te22077	TGW	22.96 ± 0.21	23.10 ± 0.16	0.0533		
		GL	8.879 ± 0.040	8.914 ± 0.036	0.0007	0.018	6.8
		GW	2.611 ± 0.011	2.614 ± 0.010	0.2334		
K5	Te22215-Te22365	TGW	22.80 ± 0.28	22.85 ± 0.40	0.5900		
		GL	8.806 ± 0.045	8.840 ± 0.052	0.0130	0.017	6.7
		GW	2.648 ± 0.027	2.641 ± 0.027	0.3870		

^a^ TGW, 1000-grain weight (g); GL, Grain length (mm); GW, Grain width (mm). ^b^ *A*, additive effect of replacing a Teqing allele with a IRBB52 allele. ^c^ *R*^2^, proportion of the phenotypic variance explained by the QTL.

**Table 2:** Annotated genes in *qGS10.2* and *qGL10.2* regions according to Nipponbare genome.

QTL	Locus Name	Gene Product Name
*qGS10.2*	*LOC_Os10g40804*	Retrotransposon protein
	*LOC_Os10g40806*	Hypothetical protein
	*LOC_Os10g40810*	GATA zinc finger domain containing protein
	*LOC_Os10g40820*	Expressed protein
	*LOC_Os10g40824*	Expressed protein
	*LOC_Os10g40830*	Metalloendoproteinase 1 precursor
	*LOC_Os10g40840*	Retrotransposon protein
	*LOC_Os10g40859*	Matrixin family protein
	*LOC_Os10g40880*	Flavonol synthase/flavanone 3-hydroxylase
	*LOC_Os10g40890*	Retrotransposon protein
	*LOC_Os10g40900*	Flavonol synthase/flavanone 3-hydroxylase
	*LOC_Os10g40920*	Pentatricopeptide
	*LOC_Os10g40934*	Flavonol synthase/flavanone 3-hydroxylase
	*LOC_Os10g40950*	Polyol transporter 5
*qGL10.2*	*LOC_Os10g41110*	Autophagy-related protein 3
	*LOC_Os10g41120*	Expressed protein
	*LOC_Os10g41130*	AP2 domain containing protein
	*LOC_Os10g41150*	Aminotransferase, classes I and II
	*LOC_Os10g41160*	Expressed protein
	*LOC_Os10g41170*	Dehydrogenase
	*LOC_Os10g41180*	Expressed protein
	*LOC_Os10g41190*	Transporter family protein
	*LOC_Os10g41200*	MYB family transcription factor
	*LOC_Os10g41220*	Protein kinase family protein
	*LOC_Os10g41224*	Expressed protein
	*LOC_Os10g41230*	Homeobox associated leucine zipper
	*LOC_Os10g41240*	Dual specificity protein phosphatase
	*LOC_Os10g41250*	Glycoprotein
	*LOC_Os10g41260*	MYB family transcription factor
	*LOC_Os10g41270*	Triacylglycerol lipase like protein
	*LOC_Os10g41280*	Expressed protein
	*LOC_Os10g41290*	AGC_PVPK_like_kin82y.17—ACG kinases include homologs to PKA, PKG and PKC
	*LOC_Os10g41300*	Expressed protein
	*LOC_Os10g41310*	DUF630/DUF632 domains containing protein
	*LOC_Os10g41320*	Hypothetical protein
	*LOC_Os10g41330*	AP2 domain containing protein

## Data Availability

The original contributions presented in the study are included in the article/Supplementary Materials, further inquiries can be directed to the corresponding author/s.

## Supplementary Materials

The following supporting information can be downloaded at: https://www.mdpi.com/article/10.3390/plants13152054/s1, Table S1: Polymorphic markers developed and used in this study.
