# Supplementary material for: Dissection and Fine-Mapping of Two QTL Controlling Grain Size Linked in a 515.6-kb Region on Chromosome 10 of Rice
Source: Plants (Basel). 2024 Jul 25;13(15):2054. doi: 10.3390/plants13152054 (PMC11314457; doi:10.3390/plants13152054)
Supplement: Supplementary file 1 [file plants-13-02054-s001.zip › plants-3090149-supplementary.pdf]

Table S1 Polymorphic markers developed and used in this study.

| Name | Tpye | Forward primer (5′-3′) | Reverse primer (5′-3′) |
|---|---|---|---|
| RM3123 | SSR | ACGCTCTTAATTGATCCGTTCG | CAAAGTCCAGTTCCGTTGATCC |
| Te21852 | InDel | TAGTATTAGTACCCGGGATTTGTGG | CAGCTAAGCATGTGAAATCAT AGTGC |
| Te21873 | InDel | GATGGATGAGAGCGGCTG | GACGCAGCGAATCCGATG |
| Te21927 | InDel | CCATGGCGAAACGAGG | ACATGCTAATACTAGGCTTTTT TTTTATAGA |
| Te21945 | InDel | TCAATCAATTCTGTTGGTATAACTATGATTG | GATGGAGAATTAACGAACGAA CGG |
| Te21986 | InDel | GAACCTGATTCAGCTTCTTTTGT | ATATAGAGACTCAAAATGCAA TAGTTTGAAAGC |
| Te21995 | InDel | AAACCACATATAAGCAGTATCCT | CTTATGCCTGTAAACTGCGTTG |
| RM25845 | SSR | TCTCGTCAGTACATGGTGGATGG | GTTCTTGAGCCAGTGCTTCTTC C |
| Te22077 | InDel | GATATTATTAATCAAAGTAGC | TTTTGAAACGATGGAAGTAGG TA |
| Te22215 | InDel | CATGTACATCGCAGGAATCCCG | TCAGGCTATTTTGAGTTACAC |
| RM228 | SSR | TCTAACTCTGGCCATTAGTCCTTGG | AAGTAGACGAGGACGACGACA GG |
| Te22365 | InDel | CACCTGCCAATACGTGAGCC | CAAAGTACAAACACGCCCGAA |
| Te22367 | InDel | TTAATTTTGTGTAGTACGGGAGA | AACAATCTTTCTTTACTCTCTA C |
| Te22984 | InDel | AACACTAATAGCAAAGCCTA | GACATATTCAGAAGCCACCC |
| RM6673 | SSR | CCACCCGTCTCATGTTCTACTCG | ATGGAGATGATACACTCGCAT CG |
